# Low- and No-Calorie Sweetener (LNCS) Consumption Patterns Amongst the Spanish Adult Population

**DOI:** 10.3390/nu13061845

**Published:** 2021-05-28

**Authors:** Marina Redruello-Requejo, María González-Rodríguez, Mª de Lourdes Samaniego-Vaesken, Ana Montero-Bravo, Teresa Partearroyo, Gregorio Varela-Moreiras

**Affiliations:** 1 Departamento de Ciencias Farmacéuticas y de la Salud, Facultad de Farmacia, Universidad San Pablo-CEU, CEU Universities, Urbanización Montepríncipe, Alcorcón, 28925 Madrid, Spain; m.redruello@usp.ceu.es (M.R.-R.); m.gonzalez202@usp.ceu.es (M.G.-R.); l.samaniego@ceu.es (M.d.L.S.-V.); amontero.fcex@ceu.es (A.M.-B.); t.partearroyo@ceu.es (T.P.); 2 Grupo USP-CEU de Excelencia “Nutrición para la vida (Nutrition for Life)”, ref: E02/0720, Alcorcón, 28925 Madrid, Spain

**Keywords:** low- and no-calorie sweeteners, foods, beverages, food additives, dietary patterns, added sugars, Spain

## Abstract

Low- and no-calorie sweeteners (LNCS) are a group of food additives characterized by their high sweetness intensity and virtually zero caloric content, attributes that make them potential substitutes for added sugars in processed foods and beverages. However, there is currently scarce information available about both the different LNCS used in food products available in Spain and their consumption patterns. Prompted by these reasons, the aim of this research work was to identify the presence and consumption of LNCS in food and beverages consumed by a representative sample of the Spanish adult population (*n* = 507). For this purpose, a Food Frequency Questionnaire was carried out. Overall, it was found that 4.5% of the foods and 22.3% of the beverages consumed by the surveyed population contained LNCS. The food groups that presented the highest percentage of daily servings containing LNCS were non-alcoholic beverages such as soft drinks and juices (36.1%); sugars and sweets such as chocolates, candies, or chewing gum (14.2%); milk and dairy products (7.0%); meat and derivative products (5.1%); cereals and derivatives (4.3%); appetizers (1.7%); and, finally, sauces and condiments such as ketchup or mustard (1.0%). The main LNCS consumed were acesulfame-K, sucralose, sorbitol, aspartame, and cyclamate, although their prevalence of use differs greatly among foods, beverages, or tabletop sweeteners. Our results show the great diversity of food groups that are currently including these compounds as ingredients. Consequently, there is a need for these food additives to be included in food composition databases, which should be regularly updated to include LNCS in order to facilitate their assessment and monitoring in dietary nutritional surveys.

## 1. Introduction

Low- and no-calorie sweeteners (LNCS) are a group of food additives characterized by their high sweetness intensity and virtually zero caloric content [1]. These attributes make them potential substitutes for added sugars in processed foods and beverages. In a context defined as the “global obesity epidemic” by the World Health Organization (WHO) [2], which back in 2003 had officially identified added sugars as one of the main dietary factors associated with the increase in overweight and obesity rates [3], the food industry has been called upon to reformulate its products in order to reduce the caloric density derived from the use of added sugars. Specifically, in Spain, the Ministry of Health, Consumer Affairs and Social Welfare, through the Spanish Agency for Food Safety and Nutrition (AESAN), has drawn up a Collaboration Plan for improvement of the composition of food and beverages that, among other measures, encourages manufacturers to reformulate and reduce the energy density and added sugar of food products [4]. The agreed reformulation measures affect food and beverages belonging to 12 groups: soft drinks, pastries and cakes, breakfast cereals, creams, meat products, cookies, ice cream, fruit nectars, special packaged bread, ready-to-eat meals, dairy products, and sauces. Therefore, the overall aim of this AESAN’s plan was to reduce, by 5 to 10% depending on the group, the food content of added sugar, saturated fats, and salt by the end of 2020. In the pursuit of this goal, LNCS have acquired a major role as total or partial substitutes for added sugars in the formulation of processed products and beverages [5,6].

By definition, LNCS are also referred to as artificial sweeteners, non-nutritive sweeteners, high intensity sweeteners, and non-caloric sweeteners. There are currently nineteen LNCS approved by the European Food Safety Authority (EFSA) for their use as food additives within Europe [7]. The authorization process for each is based on a comprehensive safety assessment that comprises hazard identification and characterization, exposure assessment, and risk characterization [8]. When possible (i.e., when sufficient information is available), an Acceptable Daily Intake (ADI) is established as the amount that is considered safe to consume each day over the course of a person’s lifetime, ideally far exceeding the estimated daily intake even for the largest consumers of this group of additives. In this way, the regulatory authorities ensure that the consumption of sweeteners is safe within these limits [9]. It should be also considered, however, that combined intakes of the vast amount of artificially sweetened products available on the market may be bringing the population’s exposure levels closer to maximum acceptable levels, and while occasionally exceeding the ADI does not necessarily pose a health risk [10], the scientific community must not be complacent about this apparent sense of security.

Prompted by the same concerns that motivated the abovementioned reformulation strategies, consumer demand for low and no-sugar products is rapidly rising [11,12]. In 2011, EFSA’s Panel on Nutrition, Novel Foods and Food Allergens (NDA Panel) evaluated the substantiation of claims related to LNCS and certain proposed beneficial health effects [1], concluding there was “no clear cause and effect relationship to substantiate the claims that intense sweeteners when replacing sugars maintain normal blood sugar levels, or maintain/achieve a normal body weight”. To date, only two claims related to LNCS have received a favorable opinion from EFSA and have therefore been authorized by the European Commission. Nonetheless, a recent systematic review commissioned by the World Health Organization (WHO) [13] concludes that “there was no compelling evidence to indicate important health benefits of non-sugar sweetener use on a range of health outcomes” and that “potential harms from the consumption of non-sugar sweeteners could not be excluded”.

Although population intake levels for added sugars are somewhat studied worldwide [14,15,16], updated information regarding LNCS intake and distribution amongst food groups is very limited in Spain [17] and, to our best knowledge, there is no monitoring or assessment of LNCS in foods and beverages sold and consumed in the country. The main impediments that account for the lack of information are, on the one hand, because European legislation on food information provided to consumers [18] requires producers to declare the use of additives as ingredients in the labeling but does not oblige them to specify the quantity used. Though this does not represent a safety problem—since additives with an assigned ADI also have an assigned maximum amount of use according to produce category—it does represent a drawback as it hinders the calculation of accurate estimates on population’s actual exposure levels. On the other, remarkably, food composition databases do not seem to reflect the rapid changes in the formulation of processed foods and beverages [19]. In Spain, all available information is virtually restricted to a publication from 2018 examining the presence and type of added sugars and LNCS in a representative sample of the food products consumed by the Spanish population from the ANIBES study [6]. On a final sample of 1164 food and beverage products, it was observed that 42% had added sugars in their composition, 10% contained LNCS, and 5% included a combination of both. It is also worth mentioning another publication from 2019 in which a novel database was created to include LNCS declared in food products marketed in Spain [20]. In addition, the “Food Consumption Survey in Spain 2019” [21] only includes explicit consumption data on tabletop sweeteners, since no classification is found in the product categories that may include sweeteners in order to address this issue. In the United States, trends in consumption of products sweetened with LNCS have been widely analyzed since 2000, concluding in a decrease in consumption of products formulated with added sugars and a corresponding increase in those with LNCS alone or combined with added sugars [22], especially by families with children [19].

There is existing controversy regarding potential harmful effects associated with the consumption of LNCS [23,24,25], despite the fact that their safety has been extensively studied by regulatory authorities and confirmed by scientific consensus documents [26]. In this current model of increasing consumption as a consequence of the continuous reformulation of many food products, a greater degree of evidence is required to help clarify the actual exposure levels and the potential risks and benefits associated with these long-term LNCS consumption levels [13]. A systematic review of available studies on LNCS intake published in 2018 [12] concluded that population intake levels were generally within the ADI levels for all these sweeteners with an acceptable degree of confidence. However, it highlighted the importance of continuing to monitor these levels due to recent initiatives implemented to reduce added sugar intake. In this regard, it should be noted that European regulations [27] include a plan for the re-evaluation of the safety of food additives that were permitted before 2009, which includes sweeteners, among others.

In accordance with the reasons motivating this study, a recent expert report in the field of low and no-calorie sweeteners in Spain (scientists, prescribers, administrations, industry, and associations), based on SWOT methodology (Strengths, Weaknesses, Opportunities, Threats) [28], identified as a weakness and as a threat, respectively, the “lack of information on actual consumption of sweeteners by the population” and the “unavailability of food composition databases for processed foods and beverages including every ingredient and additive (including sweeteners) and the fact that manufacturers are not obliged to declare the amounts added”.

Given all the above mentioned, the aim of the present work was to analyze the presence and consumption of LNCS through food and beverage products according to the consumption habits of a representative sample of the Spanish population.

## 2. Materials and Methods

### 2.1. Design of the Study

A cross-sectional descriptive observational study was designed. Each participant completed an individual survey comprising a series of questions on the profile of the individual and a food frequency questionnaire (FFQ) adapted from a validated short questionnaire on frequency of dietary intake [27]. The FFQ (supplementary material) registered the individual average consumption of 65 food and beverage items over the previous year in order to account for seasonal variation. For each processed product consumed, participants were also asked to specify, in detail, its “type” and “brand” so that product label could be consulted to analyze and register the absence or presence—and type—of LNCS. In order to guarantee the optimum design and suitability of the questionnaire to be applied, a pre-test or pilot test was carried out with the target group before proceeding to the final validation of the questionnaire and the launch of the global fieldwork.

The study protocol was approved by the Clinical Research Ethics Committee of the CEU San Pablo University, with code 447/20/27.

### 2.2. Sample

A stratified random sampling was carried out considering sample quotas according to Nielsen area, gender, and age variables. The selection of the participants was made randomly at street level, and the survey was carried out only to those people who met the inclusion criteria (people over 18 years old living in Spain with a minimum of 1 year of residence) and who agreed to sign the informed consent form. The final sample of the study, with *n* = 507 individuals, presented a sampling error of ±4.3% for a normal asymptotic confidence interval with correction for finite populations at 95.5% bilaterally and considering an infinite universe.

### 2.3. Data Collection

Fieldwork comprising the recruitment and survey processes was carried out from mid-October to December (three months) 2019 by the market research company Madison MK (TELECYL^TM^, Valladolid, Spain), while the data processing was carried out in the laboratories of the Nutrition and Food Sciences Area of the Faculty of Pharmacy of the CEU San Pablo University (Madrid, Spain). The survey was performed individually by each participant by programming the FFQ questionnaire in a Computer Aided Web Interviewing (CAWI) application to allow its online completion, with the intervention of an interviewer. This computerized data collection allowed for continuous controls and supervision based on the automatic control of the quality of the information collected in the application (response ranges allowed filters and jumps in the questions, warnings of incorrect information, etc.). In addition, a direct review of the work was carried out to supervise the quality of the information collected.

### 2.4. Assessment of LNCS Intake 

The use of the FFQ allowed for an analysis of the dietary patterns observed for the sample population, for which we estimated the daily servings consumed for each food group, and of these, the daily servings containing LNCS. In this way, the percentage of daily servings consumed with LNCS over the total daily servings consumed for each group provides us with an estimation of the extent to which these additives are included in the daily dietary habits of the Spanish population. Servings were calculated according to the recommended serving sizes included in The Spanish Society of Community Nutrition (SENC) Dietary Guidelines for the Spanish population [29].

### 2.5. Statistical Analysis

In a preliminary statistical analysis, a descriptive analysis of the sample and its main quantitative variables, expressed through centralization and dispersion parameters, was considered. To analyze the results, the Shapiro–Wilk normality test was carried out for all the samples allowing for the verification of the normality of the distribution. To verify the homogeneity of the medians, the Mann–Whitney test was performed to determine the comparisons between the medians and to detect the significantly different pairs. Finally, the Pearson chi-square test was used to verify if the frequencies observed in each category were compatible with the independence between both variables. All analyses were performed using performed with the SPSS^TM^ Statistics software, version 24 (IBM Corporation, Armonk, NY, USA). The level of statistical significance was set at *p* ≤ 0.05.

## 3. Results

The description of the sample population is included in Table 1. The final sample was made up of 507 participants, comprising 251 men and 256 women, evenly distributed within three age groups. A representative sample of Spain was ensured by using sample quotas according to the Nielsen areas. No significant differences between genders were found for the socio-demographic variables of age, education level, and geographical distribution. In terms of the occupational status, significantly greater differences were identified (*p* ≤ 0.001) in the group of retired people for men (65.7%), and in the situation of “others” for women (69.8%).

**Figure 1:**
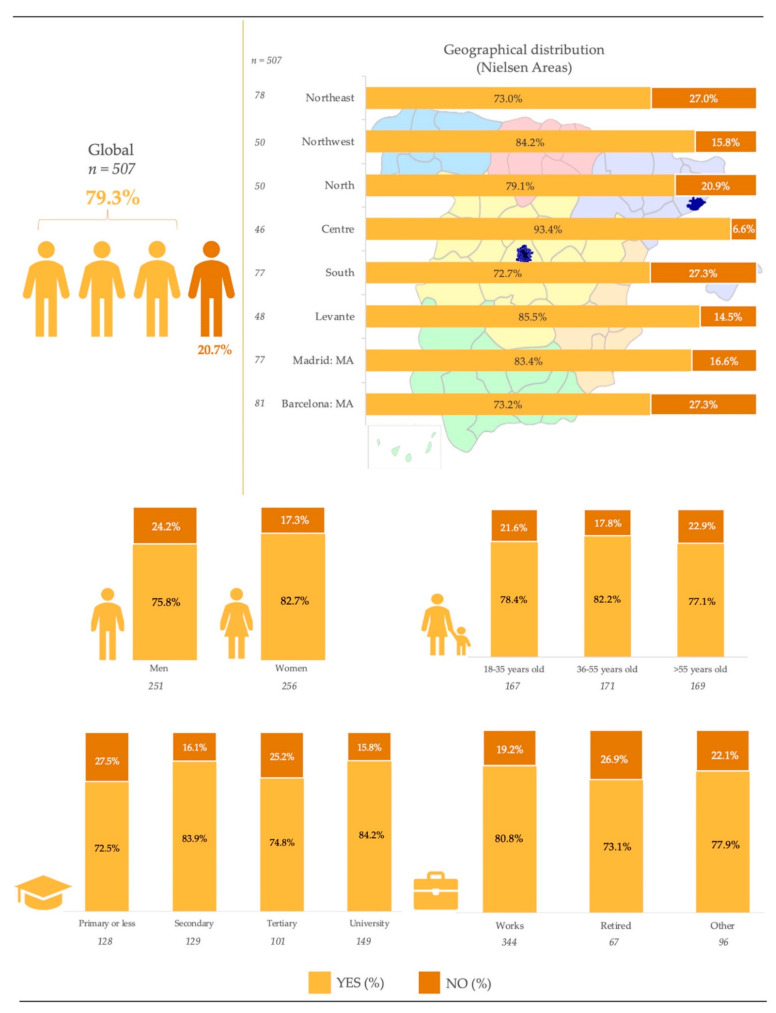
Consumption of low- and no-calorie sweeteners (LNCS) by the Spanish sample population.

Figure 1 shows the consumption of LNCS observed for the sample population, detailed for the socio-demographic variables considered. Overall, 79.3% of the total sample consume food products containing LNCS on a daily basis. The groups that consume them to a greater extent are women (82.7%), population aged 36–55 years (82.2%), those with university studies (84.2%), working (80.8%), and residents in the central area of Spain (83.4%), but no significant differences were found among any of these.

Regarding the products that accounted for the consumption of LNCS, it was found that 4.5% of the foods and 22.3% of the beverages consumed by the population contained LNCS.

Table 2 shows the consumption of LNCS from each food group expressed as the percentage of daily servings consumed with LNCS over the total daily servings consumed for each group. Food products consumed with presence of LNCS are, in decreasing order, non-alcoholic beverages (36.1%); sugars and sweets such as chewing gums, jams, and chocolates (14.2%); milk and dairy products (7.0%); processed meats (5.1%); cereals and derivatives including bakery products (4.3%); snacks (1.7%); and, finally, sauces and condiments (1.0%). In contrast, food groups for which no servings were consumed with LNCS are fresh or minimally processed foods such as eggs, fish, seafood, fruits (including canned fruits), vegetables, legumes, and nuts as well as alcoholic beverages and ready-to-eat meals such as croquettes, pastries, or pizzas.

When stratified by gender, no significant differences were observed between the percentage of servings consumed with or without LNCS, nor in their distribution, with men showing slightly higher LNCS consumption in four food groups (cereals and derivatives, appetizers, red and processed meat, and sauces and condiments) and women in the other three (milk and dairy products, sugars and sweets, and non-alcoholic beverages).

It should be noted that within the same group of foods, there is some variability in those containing LNCS in their formulation, even for quite similar products, and in this context, it is equally relevant to point out those products that did not contain LNCS. For more detailed information to understand consumption patterns, Table S1 shows not only the food groups of most relevance to LNCS intake but also the subgroups and products.

**Figure 2:**
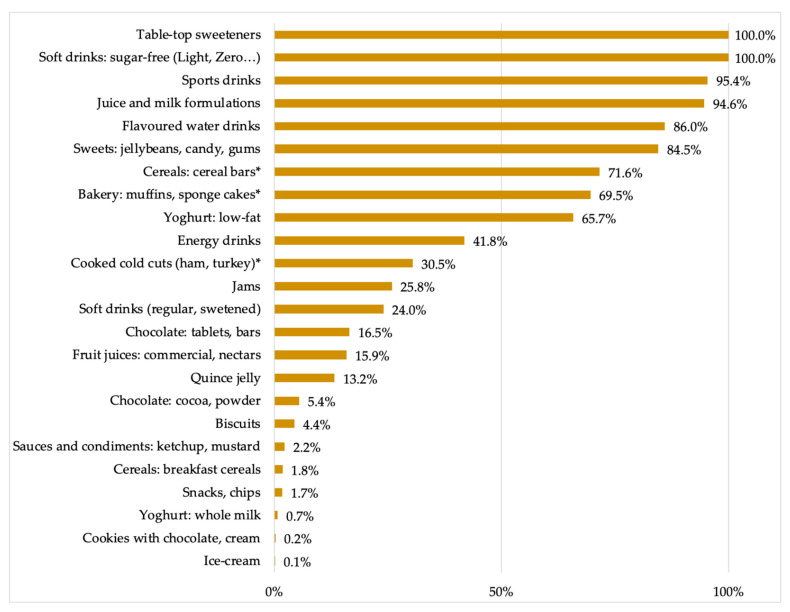
Presence of low- and-no-calorie sweeteners (LNCS) in all food products contributing to LNCS consumption. * LNCS found was sorbitol, for which the declared use was not as a sweetener but as humectant or as stabilizer.

Figure 2 details the consumption of LNCS in the food products contributing to the consumption of this group of additives. Tabletop sweeteners contributed their entire share of the servings consumed, evidencing that LNCS have displaced caloric sweeteners, which is consistent with the objective of reducing their energy contribution. Similarly, low or sugar-free soft drinks also contributed with 100% of their consumed servings as they need to include sweeteners as sugar substitutes. Following this trend are other non-alcoholic beverages such as sports drinks (95.4%), juice and milk formulations (94.6%), and flavored water drinks (86.0%). It is at this level that proper food contribution is observed, with sweets, candies and chewing gums contributing with 84.5% of their consumed servings; followed by cereal bars (71.6%), bakery products (69.5%) and low-fat yoghurts (65.7%). The remaining food products that contributed to the consumption of LNCS did so in less than 50% of their servings, indicating that at least 1 out of 2 servings consumed from these products did not contain LNCS.

Examining the presence of each individual LNCS, Table 3 displays a detailed compilation of those identified in each food product. Products including the greatest variety of LNCS in their compositions were sweets, including jellybeans, candies, and chewing gums (up to 12 different LNCS), sugar soft drinks (7 LNCS), low or no-sugar soft drinks, tabletop sweeteners, low-fat yoghurts, jams, and commercial fruit juices or nectars (6 LNCS in each). The most common LNCS present in food products consumed by the Spanish population was acesulfame K (E-950), found in the composition of 15 different food groups, followed by sucralose (E-955), present in 14, and steviol glycosides (E-960) in 10 food groups.

**Figure 3:**
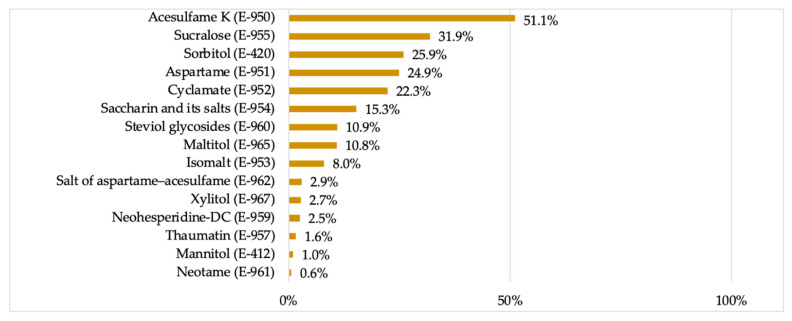
Consumption of low- and no-calorie sweeteners (LNCS) expressed as a percentage of daily servings consumed with that particular LNCS with respect to the total daily servings consumed with LNCS.

In contrast, Figure 3 shows the overall percentage of consumption of each LNCS in Spain, expressed as the percentage of daily servings consumed that contained a particular LNCS with respect to the total daily servings consumed for all foodstuffs that contained LNCS. Acesulfame-K (E-950) and sucralose (E-955) were still the most consumed LNCS, respectively present in 51.1% and 31.9% of the products consumed with this type of additives. These were then followed by sorbitol (E-420)—although sometimes used as stabilizer or as humectant, with 25.9%, and steviol glycosides (E-960) drop to the seventh position, present in 10.9% of the overall servings consumed with LNCS.

## 4. Discussion

The identification—if not the quantification—of LNCS used in foods and beverages is relevant because the European regulation establishes that their consumption must be monitored and determined in the different population groups, and this duty becomes more urgent with the constant reformulation initiatives proposed by official institutions aimed at reducing the content of added sugars in food products. To our best knowledge, this is the first work conducted in Spain aiming to provide a semi-quantitative estimate of LNCS intake in main food and beverage groups consumed by a representative sample of Spanish population. As mentioned before, due to the absence of information on the content of LNCS in the labeling of products marketed in Europe, it is simply impossible to make a direct quantitative estimate of the consumption of LNCS among its inhabitants. The present work represents a first attempt to obtain an overview of LNCS intake in Spain in a more in-depth way than the available studies that solely describe their presence across main food groups.

We analyzed LNCS intake according to daily servings consumed of each food and beverage group using individual-based consumption data (*n* = 507) obtained from a validated FFQ. In this way, the percentage of daily servings consumed with LNCS over the total daily servings consumed for each group provides some insight on the extent to which these food additives are included in the daily dietary habits of the Spanish population.

The present study confirms that LNCS are widely used across several groups of food and beverage products commercialized in Spain. As a result, more than three out of four Spanish residents consume some sort of product containing LNCS on a daily basis, with intake levels being fairly consistent for all the socio-demographic variables studied. Results on the overall high consumption levels observed confirm the great level of acceptance of this type of additive among the Spanish population, with groups exhibiting higher intakes being women (82.7%) and population aged 36–55 years (82.2%) and those with university studies (84.2%), working (80.8%), and residents in the central area (83.4%), possibly indicating the association of LNCS with modern lifestyles and diet patterns derived from individual and institutional efforts aimed to control the caloric density of the intakes and, hence, to facilitate body weight management.

Analyzing food products from which the observed consumption of LNCS is derived, since additives are only found in products with a certain degree of processing, it is not surprising to learn that it is the highly processed products that contribute the most to the consumption of LNCS, namely soft drinks, sweets and candies, pastries, and cereal bars, are the foods that largely contained LNCS, with more than 50% of the foods consumed in these categories containing some of these sweeteners in their composition.

Nonetheless, one of our main findings is that foodstuffs contributing to LNCS intake are not exclusively “light” or low-sugar products anymore, proving that the use of such additives has expanded among major food groups as a result of reformulation policies. This explains the presence of LNCS in the daily servings (%) of products that have not traditionally included these types of additives: yoghurts, both low-fat (65.7%) and even full-fat (0.7%), jams (25.8%), sugar soft drinks (24.0%), chocolate bars or tablets (16.5%), sauces like ketchup and mustard (2.2%), and snacks (1.7%). These trends are observed even in products that purposefully use added sugars as a quick source of energy, such as sports and energy drinks, which included LNCS in 95.4% and 41.8% of the servings consumed, respectively. Finally, it is also worth mentioning that the observed intake of LNCS derived from cereal bars (71.6%), bakery products such as muffins and sponge cakes (69.5%), and cooked cold cuts (30.5%) corresponds exclusively to sorbitol (E-420), for which the declared use was not as a sweetener but as a humectant or stabilizer in *quantum satis* amounts.

Consumption data obtained in the present work are consistent with a recent global review [30], where the most important contributors to LNCS consumption were found to be carbonated soft drinks, dairy (especially yoghurts), juices, and sweets as well as tabletop sweeteners. However, before we proceed with the analysis of the consumption of each individual LNCS, it is also necessary to reflect on the fact that the food groups which contribute the most to the consumption of LNCS are not necessarily those with the largest presence of LNCS among the Spanish market. For example, trends in the use of LNCS in commercially available milkshakes could be very low, yet the population could appear to have a high intake of LNCS from this food group because most people preferred to consume their light or sugar-free versions.

In order to relate consumption trends and the presence of LNCS in the food products marketed in Spain, we used the only database available in the country at the present time, the aforementioned publication from 2018, in which we studied the presence and type of added sugars and LNCS in a representative sample (*n* = 1164) of the food products consumed by the Spanish population included in the ANIBES study [6]. LNCS presence was identified in 39% of non-alcoholic beverages (vs. 36.1% daily servings consumed with LNCS in the present study); 15% of the sugars and sweets group (vs. 14.2% in this study); 12% of dairy and derivatives (vs. 7.0% in the present study); 3% of meats and derivatives (vs. 5.1% in this work); or 5% of cereals and derivatives including pastries (vs. 4.3% in the present work). It may be surprising to learn how the presence of LNCS in the different food groups available in the Spanish market almost exactly matches the contribution of each group to the consumption of LNCS observed for the population surveyed in this study. This coincidence may be explained by considering that the food industry, like any other market, invariably examines the consumption trends of consumers to adapt to their preferences and thus maintain the competitiveness of the offered products. In addition, another cause of this parity in presence and consumption data could be one of the main limitations found when using the FFQ, where some of the participants stated that they did not pay attention to the labeling of the products, regardless of whether they had some additives or others, or even if they had sugars or not. Thus, the probability of choosing a product with sweeteners is mostly determined by the frequency of use of sweeteners in that type of product. This observation reinforces the evidence on the need to monitor the levels of exposure of the population to LNCS, even more so when considering that recent reformulation efforts may not only increase the content of LNCS in products that already contained them but continue to expand the range of products that begin to include these types of additives.

In this regard, it is worth noting some recent evaluations on the intake of LNCS in Chile, after enforcement of a food labeling law that regulates added sugar content in processed foods. In an interesting approach, the Chilean regulation states that food labeling must indicate the amount of these additives per serving and for 100 g or 100 mL of the product ready for consumption along with the ADI in mg/kg body weight for each LNCS, which allows for a more accurate analysis of food composition trends with regards to these additives. In a very illustrative example of how quickly food formulations and products can evolve, one study reports that steviol glycosides, just eight years after first appearing in the Chilean market in 2009, are nowadays present in 21.8% of all food products [31]. Fortunately, these rapid increases in LNCS use do not appear to exceed safety exposure levels at the moment, with another study from 2020 reporting a “large, but admissible, exposure to sugar substitutes among schoolchildren living in the city of Santiago” [32]. In any case, the information available on Chile is of great interest since it is one of the countries with the highest proportions of LNCS-containing food products, with a total of 55.5% compared to 7.7% for Spain in 2019 [31].

Proceeding with the individual analysis of each LNCS, it has already been mentioned that the present study aimed to analyze the consumption of LNCS by the Spanish resident population, rather than their mere presence across the Spanish market, and indeed some differences were found between the two variables. Acesulfame K (E-950) and sucralose (E-955) were both the most present and consumed ones, respectively found in the composition of 15 and 14 different groups of foods each and in 51.1% and 31.9% of the foodstuffs consumed with this type of additive. However, steviol glycosides were found to be the third most common LNCS across all food groups (present in 10 of them), yet their consumption drops to the seventh position with 10.9% of the overall servings consumed with LNCS.

It is noteworthy that those LNCS that are best known to the general public, such as saccharin or steviol glycosides, are not at the top of this list, respectively present in 15.3% and 10.9% of LNCS-containing products in the present study and 6.1% and 1.1% in the ANIBES [6]. As already discussed, the predominance on the use of one or another LNCS varies widely between food categories, and only in the case of tabletop sweeteners is the consumer more aware of the substance they are consuming and are therefore more familiar with it. In fact, it is within the group of tabletop sweeteners where the predominant use of saccharin is observed, followed by cyclamate and steviol glycosides. Notably, 100% of the tabletop sweeteners consumed were LNCS, having displaced caloric sweeteners in a consistent approach with the objective of reducing their energy contribution.

In the perspective of these results, the great variability of exposure to each LNCS according to the category of food product from which this consumption is made should be highlighted. In this regard, the aforementioned expert report [28] states that “although globally there is evidence on the safety of LNCS in common situations, the abuse of these products and the effect of their long-term consumption should be studied individually for each compound used as a sweetener”. This should be taken into account to avoid attributing the characteristics or scientific evidence of one of them to the other eighteen. For example, one of the growing concerns about the use of LNCS would be their possible altering effects on intestinal microbiota, and it should be noted that the available evidence [23] indicates that such effects would be restricted exclusively to saccharin, sucralose, steviol glycosides, and polyols (erythritol, isomalt, polyglycitol syrup, lactitol, maltitol, mannitol, sorbitol, and xylitol). Moreover, in the case of polyols, such alterations would not be harmful but potentially beneficial as they behave as prebiotic agents.

Combined analysis on the intake of LNCS derived both from food and beverages is one of the main strengths of the present work. In addition, a thorough analysis of the nutrition labeling of each product reported by the sample population added reliability and representativity to the data collection and compensates for one of the main limitations encountered when using the FFQ, where some of the participants reported not paying attention to product labeling. However, we cannot overlook other limitations inherent in the use of dietary assessments such as underreporting derived from participant and recall biases. Other strengths that have enabled us to provide a comprehensive overview of LNCS consumption across the country are the inclusion of a large nationally representative sample (*n* = 507) that includes all Spanish Nielsen Geographical Areas and different age segments.

## 5. Conclusions

The present work confirms that LNCS are widely used across several groups of food and beverage products commercialized in Spain. As a result, 8 out of 10 inhabitants of the country have been estimated to consume food products containing LNCS on a daily basis, with intake levels being fairly consistent for all the socio-demographic variables studied.

In particular, acesulfame K (E-950), sucralose (E-955), sorbitol (E-420), aspartame (E-951), and cyclamate (E-952) were found to be the most consumed LNCS within the sample population. However, there is a great variability in the prevalence of use for each LNCS according to food category: foods, beverages, or tabletop sweeteners. Consequently, there is a great variability of exposure to each LNCS according to the product from which this consumption is derived.

Nonetheless, it seems necessary to draw attention to the large number of LNCS blends contained in some foodstuffs. There is currently little evidence of the potential effects of these combinations—even less so in the long term and at the current increasing exposure levels—and yet products formulated with LNCS but not requiring combinations of these appear to be a clear minority.

By all means, the present work evidences the need for such food additives to be compiled in food composition databases, which should be periodically updated to include LNCS and facilitate their evaluation and monitoring in nutritional surveys. This is even more so the case when considering that the constant reformulation strategies implemented to reduce the consumption of added sugars are mostly based on their replacement with LNCS.

## Figures and Tables

**Table 1:** General description of the sample population.

		** 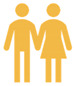 **		** 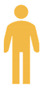 **		** 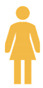 **	
		***n***	**%**	***n***	**%**	***n***	**%**
**Global**		507	100	251	49.5	256	50.5
** 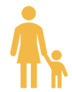 **	18–35 years	167	33.0	86	51.5	81	48.5
	36–55 years	171	33.7	80	46.8	91	53.2
	>55 years	169	33.3	85	50.3	84	49.7
** 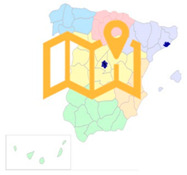 **	Northeast	78	15.4	39	50.0	39	50.0
	Northwest	50	9.9	25	50.0	25	50.0
	North	50	9.9	23	46.0	27	54.0
	Centre	46	9.1	24	52.2	22	47.8
	South	77	15.1	40	51.9	37	48.1
	Levante	48	9.5	23	47.9	25	52.1
	Madrid: MA ^(1)^	77	15.2	38	49.4	39	50.6
	Barcelona: MA ^(1)^	81	16.0	39	48.1	42	51.9
** 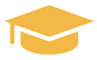 **	Primary or less	128	25.2	67	52.3	61	47.7
	Secondary	129	25.4	58	45.0	71	55.0
	Tertiary	101	19.9	51	50.5	50	49.5
	University	149	29.4	75	50.3	74	49.7
** 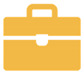 **	Works	344	67.9	177	51.7	167	48.3
	Retired	67	13.2	44	65.7 *	23	34.3
	Other: unemployed, students, housewives	96	18.9	29	30.2 *	67	69.8

^(1)^ MA: Metropolitan area. * *p* ≤ 0.001 with respect to women (Pearson’s chi-squared test).

**Table 2:** Presence of low- and-no-calorie sweeteners (LNCS) in food groups consumed by the sample population.

	Presence of LNCS	
Food Group	Yes (%)	No (%)
Appetizers	1.7	98.3
Beverages: alcoholic	0.0	100.0
Beverages: non-alcoholic	36.1	63.9
Canned fruit	0.0	100.0
Cereals and derivatives	4.3	95.7
Eggs	0.0	100.0
Fish and shellfish	0.0	100.0
Fruits	0.0	100.0
Meat: red and/or processed	5.1	94.9
Milk and dairy products	7.0	93.0
Nuts and seeds	0.0	100.0
Pulses	0.0	100.0
Ready-to-eat meals	0.0	100.0
Sauces and condiments	1.0	99.0
Sugar and sweets	14.2	85.9
Vegetables	0.0	100.0

**Table 3:** Presence and type of low- and-no-calorie sweeteners (LNCS) in all food products contributing to LNCS consumption.

Food Product	%	Nº	LNCS	Food Product	%	Nº	LNCS
Table top sweeteners	100.0	6	Acesulfame K (E-950) Aspartame (E-951) Cyclamate (E-952) Saccharin and its salts (E-954) Steviol glycosides (E-960) Thaumatin (E-957)	Soft drinks: Regular (sweetened)	24.0	7	Acesulfame K (E-950) Aspartame (E-951) Cyclamate (E-952) Steviol glycosides (E-960) Neohesperidine-DC (E-959) Saccharin and its salts (E-954) Sucralose (E-955)
Soft drinks: Low or sugar-free (Light, Diet, Zero…)	100.0	6	Acesulfame K (E-950) Aspartame (E-951) Cyclamate (E-952) Saccharin and its salts (E-954) Steviol glycosides (E-960) Sucralose (E-955)	Chocolate: tablets, bars	16.5	2	Steviol glycosides (E-960) Maltitol (E-965)
Sports drinks	95.4	3	Acesulfame K (E-950) Aspartame (E-951) Sucralose (E-955)	Fruit juices: commercial, nectars	15.9	6	Acesulfame K (E-950) Cyclamate (E-952) Neohesperidine-DC (E-959) Saccharin and its salts (E-954) Steviol glycosides (E-960) Sucralose (E-955)
Juice and milk formulations	94.6	2	Acesulfame K (E-950) Sucralose (E-955)	Quince jelly	13.2	3	Maltitol (E-965) Steviol glycosides (E-960) Sucralose (E-955)
Flavoured water drinks	86.0	4	Acesulfame K (E-950) Isomalt (E-953) Steviol glycosides (E-960) Sucralose (E-955)	Chocolate: cocoa, powder	5.4	2	Acesulfame K (E-950) Salt of aspartame-acesulfame K (E-962)
Sweets: jelly beans, candy, chewing gum	84.5	12	Acesulfame K (E-950) Aspartame (E-951) Isomalt (E-953) Maltitol (E-965) Mannitol (E-421) Neohesperidine-DC (E-959) Saccharin and its salts (E-954) Salt of aspartame-acesulfame K (E-962) Sorbitol (E-420) Steviol glycosides (E-960) Sucralose (E-955) Xylitol (E-967)	Biscuits	4.4	3	Isomalt (E-953) Maltitol (E-965) Sucralose (E-955)
Cereals: cereal bars	71.6	1	Sorbitol (E-420) *	Sauces and condiments: ketchup, mustard	2.2	3	Cyclamate (E-952) Saccharin and its salts (E-954) Sucralose (E-955)
Bakery: muffins, sponge cakes	69.5	1	Sorbitol (E-420) *	Cereals: breakfast cereals	1.8	2	Acesulfame K (E-950) Maltitol (E-965)
Yoghurt: low-fat	65.7	6	Acesulfame K (E-950) Aspartame (E-951) Neotame (E-961) Salt of aspartame-acesulfame K (E-962) Steviol glycosides (E-960) Sucralose (E-955)	Appetizers: snacks, chips	1.7	1	Aspartame (E-951)
Energy drinks	41.8	3	Acesulfame K (E-950) Aspartame (E-951) Sucralose (E-955)	Yoghurt: whole milk	0.7	3	Acesulfame K (E-950) Neotame (E-961) Sucralose (E-955)
Cooked cold cuts (ham, turkey)	30.5	1	Sorbitol (E-420) *	Cookies: with chocolate, cream	0.2	1	Maltitol (E-965)
Jams	25.8	6	Acesulfame K (E-950) Aspartame (E-951) Steviol glycosides (E-960) Maltitol (E-965) Sorbitol (E-420) Sucralose (E-955)	Ice-cream	0.1	2	Acesulfame K (E-950) Maltitol (E-965)

* LNCS found was sorbitol, for which the declared use was not as a sweetener but as humectant or as stabilizer. %: Percentage of food products containing any LNCS; Nº: number of distinct LNCS found in each food subgroup.

## Data Availability

The data presented in this study are available on request from the corresponding author.

## Supplementary Materials

The following are available online at https://www.mdpi.com/article/10.3390/nu13061845/s1, Table S1. Food groups contributing to Low- and no-calorie sweeteners (LNCS) consumption: detailed list of food products consumed with and without LNCS.
