# Supplementary material for: Low- and No-Calorie Sweetener (LNCS) Consumption Patterns Amongst the Spanish Adult Population
Source: Nutrients. 2021 May 28;13(6):1845. doi: 10.3390/nu13061845 (PMC8230335; doi:10.3390/nu13061845)
Supplement: Supplementary file 1 [file nutrients-13-01845-s001.zip › nutrients-1189132-supplementary.pdf]

Table S1. Food groups contributing to Low- and no-calorie sweeteners (LNCS) consumption: detailed list of food products consumed with and without LNCS.

**Food group**	**Food products consumed with LNCS**	**Food products consumed without LNCS**
**Food groups advised for daily or weekly consumption:**		
Cereals and derivatives	Bakery: muffins, sponge cake<br>Biscuits and cookies<br>Cereals: breakfast, bars	Bakery: croissants, doughnuts<br>Bread<br>Cakes: chocolate, cream pies<br>Pasta<br>Rice
Milk and dairy products	Ice-cream<br>Yoghurt: all types	Dairy desserts: custard, flan, rice pudding<br>Cheese: all types<br>Milk: all types<br>Milk: fermented (kefir)<br>Milkshakes
**Food groups advised for an optional, occasional, and moderate consumption:**		
Appetizers	Snacks	-
Beverages:<br>non-alcoholic	Energy drinks<br>Flavoured water drinks<br>Juices: commercial, nectars<br>Juice and milk formulations<br>Soft drinks: all types<br>Sports drinks	Juices: natural<br>Tigernut milk ("*horchata*")<br>Vegetable milk
Meat:<br>red and/or processed	Cold cuts: cooked, ham, turkey	Beef, pork, lamb<br>Cold cuts: "*Serrano*" ham<br>Minced meat, sausages, burgers
Sugar and sweets	Chocolate: all types<br>Jam<br>Quince jelly<br>Sweets: jellybeans, candy, chewing gum	Table sugar
Sauces and condiments	Ketchup, mustard	Fried tomato sauce

FOOD FREQUENCY QUESTIONNAIRE

1.- To fill in the following table, in the case of consuming different brands or varieties of each food item, please insert as many lines as necessary in order to detail every consumed product.

**FOOD ITEMS**	**FOR HOW MANY MONTHS?**	**HOW OFTEN CONSUMED?**			**PRODUCT INFO**<br>-Brand<br>-Variety<br>-Additional information regarding sugar and/or sweetener content
		**DAILY**	**WEEKLY**	**MONTHLY**	
**Whole milk**<br>(one glass)	a) Never or hardly ever: proceed to next food item.<br>b) Some months of the year: record how many.<br>c) Every month.				
**Semi-skimmed milk**<br>(one glass)					
**Skimmed milk**<br>(one glass)					
**Whole milk yoghurt**<br>(one cup)					
**Low-fat yoghurt**<br>(one cup)					
**Milkshakes**<br>(one glass)					
**Chocolate:**<br>**tablets** (two squares), **chocolates** (one piece)					
**Vegetable milk**<br>(one glass)					
**Fermented milk, kefir** (one glass)					
**Tigernut milk**<br>(one glass)					
**Breakfast cereals**<br>(one bowl)					
**Biscuits, “María” style**<br>(4 biscuits)					
**Cookies with chocolate or cream**<br>(4 cookies)					
**Muffins** (one unit),<br>**sponge cake** (one piece)					
**Croissant, doughnut**<br>(one unit)					
**Cereal bars**<br>(one unit)					

OBSERVATIONS:

1.- To fill in the following table, please indicate the consumption of each group. For example, if you normally eat noodles one day a week, macaroni one day a week and spaghetti one day a week, you will have to indicate three days a week in the group (pasta: noodles, macaroni, spaghetti...).
For food items where PRODUCT INFO is shaded in grey, it is only necessary to record frequency of consumption, not brand or variety.

FOOD ITEMS	FOR HOW MANY MONTHS?	HOW OFTEN CONSUMED?			PRODUCT INFO<br>-Brand<br>-Variety<br>-Additional information regarding sugar and/or sweetener content
		DAILY	WEEKLY	MONTHLY	
**Salad: lettuce, tomato, endive, etc.**<br>(one portion)	a) Never or hardly ever: proceed to next food item.<br>b) Some months of the year: record how many.<br>c) Every month.				
**Green beans, chard or spinach**<br>(one garnish)					
**Aubergine, mushrooms**<br>(one garnish)					
**Fried tomato**<br>(one portion)					
**Potatoes: baked, fried or boiled**<br>(one garnish)					
**Pulses: lentils, chickpeas, beans, etc.**<br>(one plate)					
**Rice: white, “paella”, etc.**<br>(one plate)					
**Pasta: noodles, macaroni, spaghetti, etc.**<br>(one plate)					
**Soups and creams**<br>(one bowl)					
OBSERVATIONS:					

1.- To fill in the following table, please indicate the consumption of each group. For example, if you normally eat pork one day a week and veal one day a week, you will have to indicate two days a week in the group (Veal, pork, lamb (steak, pie...).

For food items where PRODUCT INFO is shaded in grey, it is only necessary to record frequency of consumption, not brand or variety.

FOOD ITEMS	FOR HOW MANY MONTHS?	HOW OFTEN CONSUMED?			PRODUCT INFO<br>-Brand<br>-Variety<br>-Additional information regarding sugar and/or sweetener content
		DAILY	WEEKLY	MONTHLY	
**Eggs**<br>(one)	a) Never or hardly ever: proceed to next food item.<br>b) Some months of the year: record how many.<br>c) Every month.				
**Chicken or turkey**<br>(one portion)					
**Beef, pork, lamb (steak, pie, etc.)**<br>(one portion)					
**Minced meat, burgers, sausages**<br>(one portion)					
**White fish: hake, grouper, etc.**<br>(one portion)					
**Blue fish: sardines, tuna, salmon, etc.**<br>(one portion)					
**Canned fish**<br>(one can)					
**Seafood: mussels, prawns, king prawns, squid, etc.**<br>(one portion)					
**Canned seafood**<br>(one can)					
**Croquettes, pasties, pizza**<br>(one portion)					
**Bread**<br>(two slices)					

OBSERVATIONS:

1.- To fill in the following table, in the case of consuming different brands or varieties of each food item, please insert as many lines as necessary in order to detail every consumed product.

**FOOD ITEMS**	**FOR HOW MANY MONTHS?**	**HOW OFTEN CONSUMED?**			**PRODUCT INFO**<br>-Brand<br>-Variety<br>-Additional information regarding sugar and/or sweetener content
		**DAILY**	**WEEKLY**	**MONTHLY**	
**Cold cuts, turkey ham, “serrano” ham**<br>(two slices)	a) Never or hardly ever: proceed to next food item.<br>b) Some months of the year: record how many.<br>c) Every month.				
**Cured sausages, “longaniza”**<br>(two units)					
**White or fresh cheese (Burgos, etc.) or light cheese**<br>(one portion)					
**Other cheeses: cured or semi-cured, creamy**<br>(one portion)					

<u>OBSERVATIONS:</u>

1.- To fill in the following table, in the case of consuming different brands or varieties of each food item, please insert as many lines as necessary in order to detail every consumed product.
For food items where PRODUCT INFO is shaded in grey, it is only necessary to record frequency of consumption, not brand or variety.

FOOD ITEMS	FOR HOW MANY MONTHS?	HOW OFTEN CONSUMED?			**PRODUCT INFO**<br>-Brand<br>-Variety<br>-Additional information regarding sugar and/or sweetener content
		DAILY	WEEKLY	MONTHLY	
**Fresh fruit**<br>(one piece)	a) Never or hardly ever: proceed to next food item.<br>b) Some months of the year: record how many.<br>c) Every month.				
**Canned fruit (in syrup, etc.)** (one piece or portion)					
**Quince jelly**<br>(one portion)					
**Natural fruit juices**<br>(one glass)					
**Commercial fruit juices**<br>(one glass)					
**Juice and milk beverages**<br>(one glass)					
**Nuts: peanuts, hazelnuts, almonds, etc.**<br>(a handful)					
**Dairy desserts: custard, flan, cottage cheese, rice pudding**<br>(one portion)					
**Cakes, cream or chocolate**<br>(one piece)					
**Sweets: jelly beans, sweets, chewing gum**<br>(one unit)					
**Bags of snacks, chips**<br>(one portion)					
**Ice cream**<br>(one unit)					
**Jam**<br>(1 teaspoon)					

OBSERVATIONS:

1.- To fill in the following table, in the case of consuming different brands or varieties of each food item, please insert as many lines as necessary in order to detail every consumed product.
For food items where PRODUCT INFO is shaded in grey, it is only necessary to record frequency of consumption, not brand or variety.

FOOD ITEMS	FOR HOW MANY MONTHS?	HOW OFTEN CONSUMED?			**PRODUCT INFO**<br>-Brand<br>-Variety<br>-Additional information regarding sugar and/or sweetener content
		DAILY	WEEKLY	MONTHLY	
**Flavoured water**<br>(one glass)	a) Never or hardly ever: proceed to next food item.<br>b) Some months of the year: record how many.<br>c) Every month.				
**Sugar soft drinks**<br>(one glass)					
**Sugar-free soft drinks (Light, Zero, etc.)**<br>(one glass)					
**Sports drinks**<br>(one glass)					
**Energy drinks**<br>(one glass)					
**Wine, "sangría"**<br>(one glass)					
**Beer**<br>(one drink)					
**Distilled beverages: whisky, gin, cognac, etc.**<br>(one drink)					
OBSERVATIONS:					

1.- To fill in the following table, in the case of consuming different brands or varieties of each food item, please insert as many lines as necessary in order to detail every consumed product.

FOOD ITEMS	FOR HOW MANY MONTHS?	HOW OFTEN CONSUMED?			PRODUCT INFO<br>-Brand<br>-Variety<br>-Additional information regarding sugar and/or sweetener content
		DAILY	WEEKLY	MONTHLY	
**Ready-to-eat meals**<br>(one portion)	a) Never or hardly ever: proceed to next food item.<br>b) Some months of the year: record how many.<br>c) Every month.				
**Sauces and condiments, Ketchup, mustard, mayonnaise, etc.**<br>(one tablespoon)					
**Table sugar***<br>(one teaspoon or sachet)					
**Table-top sweeteners***<br>(one teaspoon or sachet)					
OBSERVATIONS:					

***Note:**

- If you drink coffee, and you add sugar or other sweetener to it, please indicate under 'table sugar' or 'sweeteners'.
- If you eat biscuits, cakes or other homemade baked goods and add sugar or other sweeteners, please indicate under 'table sugar' or 'sweeteners'.
